# Sociodemographic, Clinical, and Treatment-Related Factors Associated With Hyperglycemic Crises Among Adults With Type 1 or Type 2 Diabetes in the US From 2014 to 2020

**DOI:** 10.1001/jamanetworkopen.2021.23471

**Published:** 2021-09-01

**Authors:** Rozalina G. McCoy, Rodolfo J. Galindo, Kavya Sindhu Swarna, Holly K. Van Houten, Patrick J. O’Connor, Guillermo E. Umpierrez, Nilay D. Shah

**Affiliations:** 1 Division of Community Internal Medicine, Department of Medicine, Mayo Clinic, Rochester, Minnesota; 2 Mayo Clinic Robert D. and Patricia E. Kern Center for the Science of Health Care Delivery, Rochester, Minnesota; 3 Division of Endocrinology, Department of Medicine, Emory University School of Medicine, Grady Memorial Hospital, Atlanta, Georgia; 4 HealthPartners Institute Center for Chronic Care Innovation, Minneapolis, Minnesota; 5 OptumLabs, Eden Prairie, Minnesota

## Abstract

**Importance:**

Hyperglycemic crises (ie, diabetic ketoacidosis [DKA] and hyperglycemic hyperosmolar state [HHS]) are life-threatening acute complications of diabetes. Efforts to prevent these events at the population level have been hindered by scarce granular data and difficulty in identifying individuals at highest risk.

**Objective:**

To assess sociodemographic, clinical, and treatment-related factors associated with hyperglycemic crises in adults with type 1 or type 2 diabetes in the US from 2014 to 2020.

**Design, Setting, and Participants:**

This retrospective cohort study analyzed administrative claims and laboratory results for adults (aged ≥18 years) with type 1 or type 2 diabetes from the OptumLabs Data Warehouse from January 1, 2014, through December 31, 2020.

**Main Outcomes and Measures:**

Rates of emergency department or hospital visits with a primary diagnosis of DKA or HHS (adjusted for age, sex, race/ethnicity, and region, and for year when calculating annualized rates) were calculated separately for patients with type 1 diabetes and type 2 diabetes. The associations of sociodemographic factors (age, sex, race/ethnicity, region, and income), clinical factors (comorbidities), and treatment factors (glucose-lowering medications, hemoglobin A_1c_) with DKA or HHS in patients with type 1 or type 2 diabetes were assessed using negative binomial regression.

**Results:**

Among 20 156 adults with type 1 diabetes (mean [SD] age, 46.6 [16.5] years; 51.2% male; 72.6% White race/ethnicity) and 796 382 with type 2 diabetes (mean [SD] age, 65.6 [11.8] years; 50.3% female; 54.4% White race/ethnicity), adjusted rates of hyperglycemic crises were 52.69 per 1000 person-years (95% CI, 48.26-57.12 per 1000 person-years) for type 1 diabetes and 4.04 per 1000 person-years (95% CI, 3.88-4.21 per 1000 person-years) for type 2 diabetes. In both groups, factors associated with the greatest hyperglycemic crisis risk were low income (≥$200 000 vs <$40 000: type 1 diabetes incidence risk ratio [IRR], 0.61 [95% CI, 0.46-0.81]; type 2 diabetes IRR, 0.69 [95% CI, 0.56-0.86]), Black race/ethnicity (vs White race/ethnicity: type 1 diabetes IRR, 1.33 [95% CI, 1.01-1.74]; type 2 diabetes IRR, 1.18 [95% CI, 1.09-1.27]), high hemoglobin A_1c_ level (≥10% vs 6.5%-6.9%: type 1 diabetes IRR, 7.81 [95% CI, 5.78-10.54]; type 2 diabetes IRR, 7.06 [95% CI, 6.26-7.96]), history of hyperglycemic crises (type 1 diabetes IRR, 7.88 [95% CI, 6.06-9.99]; type 2 diabetes IRR, 17.51 [95% CI, 15.07-20.34]), severe hypoglycemia (type 1 diabetes IRR, 2.77 [95% CI, 2.15-3.56]; type 2 diabetes IRR, 4.18 [95% CI, 3.58-4.87]), depression (type 1 diabetes IRR, 1.62 [95% CI, 1.37-1.92]; type 2 diabetes IRR, 1.46 [95% CI, 1.34-1.59]), neuropathy (type 1 diabetes IRR, 1.64 [95% CI, 1.39-1.93]; type 2 diabetes IRR, 1.25 [95% CI, 1.17-1.34]), and nephropathy (type 1 diabetes IRR, 1.22 [95% CI, 1.01-1.48]; type 2 diabetes IRR, 1.23 [95% CI, 1.14-1.33]). Age had a U-shaped association with hyperglycemic crisis risk in patients with type 1 diabetes (compared with patients aged 18-44 years: 45-64 years IRR, 0.72 [95% CI, 0.59-0.87]; 65-74 years IRR, 0.62 [95% CI, 0.47-0.80]; ≥75 years IRR, 0.96 [95% CI, 0.66-1.38]). In type 2 diabetes, risk of hyperglycemic crises decreased progressively with age (45-64 years IRR, 0.57 [95% CI, 0.51-0.63]; 65-74 years IRR, 0.44 [95% CI, .39-0.49]; ≥75 years IRR, 0.41 [95% CI, 0.36-0.47]). In patients with type 2 diabetes, higher risk was associated with sodium-glucose cotransporter 2 inhibitor therapy (IRR, 1.30; 95% CI, 1.14-1.49) and insulin dependency (compared with regimens with bolus insulin: regimens with basal insulin only, IRR, 0.69 [95% CI, 0.63-0.75]; and without any insulin, IRR, 0.36 [95% CI, 0.33-0.40]).

**Conclusions and Relevance:**

In this cohort study, younger age, Black race/ethnicity, low income, and poor glycemic control were associated with an increased risk of hyperglycemic crises. The findings suggest that multidisciplinary interventions focusing on groups at high risk for hyperglycemic crises are needed to prevent these dangerous events.

## Key Points

**Question:**

What factors are associated with the occurrence of hyperglycemic crises (diabetic ketoacidosis and hyperglycemic hyperosmolar state) among adults with diabetes?

**Findings:**

In this cohort study of 816 538 adults with diabetes in the US (20 156 adults with type 1 diabetes and 796 382 with type 2 diabetes), younger age, low income, Black race/ethnicity, high hemoglobin A_1c_ level, prior hyperglycemic crises, severe hypoglycemia, depression, neuropathy, and nephropathy were associated with increased risk of hyperglycemic crises in both groups.

**Meaning:**

The findings suggest that multidisciplinary interventions focusing on groups at high risk of hyperglycemic crises are needed to decrease the incidence and impact of these potentially preventable complications of diabetes.

## Teaser

This cohort study uses administrative claims and laboratory results from a nationwide database to assess the sociodemographic, clinical, and treatment-related factors associated with diabetic ketoacidosis and hyperglycemic hyperosmolar states among adults with type 1 or type 2 diabetes in the US from 2014 to 2020.

## Introduction

More than 1 in 8 American adults are living with diabetes.^1^ Diabetic ketoacidosis (DKA) and hyperglycemic hyperosmolar state (HHS)^2^ are life-threatening diabetes emergencies^3^ that are associated with substantial morbidity,^4^ mortality,^5,6,7^ high costs,^8,9^ and health care use.^5,10^ Although several studies have examined factors associated with DKA or HHS in the general population,^11,12,13,14^ there is limited contemporary evidence on patient-level risk factors, hindering efforts to proactively identify patients at risk and to reduce the frequency of these events at the population level.

Most published research has focused on DKA in patients with type 1 diabetes, often among children or young adults.^15^ The frequently cited factors associated with DKA in this context are adolescence, socioeconomic disadvantage, female sex, elevated hemoglobin A_1c_ (HbA_1c_) level, prior DKA, and comorbid mental health conditions.^15^ A recent population-based analysis found that rates of DKA and HHS approximately doubled between 2009 and 2015,^16^ with higher rates among younger people and individuals residing in lower-income areas.^16^ However, these studies were limited by the lack of contemporary, longitudinal, patient-level data and granular information that differentiates patients by diabetes type, considers both type 1 diabetes and type 2 diabetes, and examines both DKA and HHS hyperglycemic crises. Accordingly, we examined emergency department (ED) visits and hospitalizations for hyperglycemic crises among adults with type 1 diabetes or type 2 diabetes in the US between 2014 and 2020, focusing on patient-level sociodemographic, clinical, and treatment-related factors associated with these events.

## Methods

### Study Design

This retrospective cohort study used medical and pharmacy claims data from OptumLabs Data Warehouse (OLDW), a deidentified claims database for privately insured and Medicare Advantage enrollees in a nationwide private US health plan.^17^ The OLDW database contains longitudinal health information on enrollees, representing a diverse mix of ages, races/ethnicities, and geographic regions across the US. All study data were deidentified in accordance with Health Insurance Portability and Accountability Act expert deidentification determination.^18^ Mayo Clinic, Rochester, Minnesota, deemed this study to be exempt from institutional review board review and no informed consent was required (or feasible) because all data were deidentified. The results are reported in accordance with the Strengthening the Reporting of Observational Studies in Epidemiology (STROBE) guideline.^19^

### Study Population

We identified adults (age ≥18 years) with diabetes included in OLDW with available HbA_1c_ data between January 1, 2014, and December 31, 2019 (index date), and 1 year of uninterrupted insurance coverage before that date. The diagnosis of diabetes was established using Healthcare Effectiveness Data and Information Set criteria^20^ and categorized as type 1 diabetes or type 2 diabetes as previously described.^11,21,22^

### Outcomes

The primary outcome was ED visit or hospitalization with a primary or first diagnosis of DKA or HHS (eTable 1 in the Supplement) that occurred between January 1, 2014, and December 31, 2020. In a secondary analysis, DKA and HHS were considered separately; in the event that both codes were present on the same encounter, events were classified as DKA in patients with type 1 diabetes and as HHS in patients with type 2 diabetes.

### Independent Variables

Patient age, sex, race/ethnicity, annual household income, and US census region of residence were identified from OLDW enrollment files at the index date. Comorbidities were ascertained from all claims during the 12 months preceding the index date as described in eTable 1 in the Supplement.

Glucose-lowering therapy was characterized based on prescriptions filled during the 120 days before the index date. For patients with type 1 diabetes, we assessed whether they had any prescriptions filled for noninsulin medication(s), prescriptions filled for insulin without noninsulin medications, or no prescriptions filled. For patients with type 2 diabetes, we first assessed whether there were any prescriptions filled, followed by whether there were prescriptions filled for bolus insulin (with or without basal insulin), prescriptions filled for basal insulin, or no prescriptions filled for insulin. Then, we identified fills for individual classes of noninsulin medications (eTable 2 in the Supplement).

### Statistical Analysis

We assessed overall frequencies (percentages) and means (SDs) for baseline patient characteristics using the Mantel-Haenszel χ^2^ test for categorical variables and the *t* test for continuous variables. Crude and adjusted rates of ED visits or hospitalizations for hyperglycemic crises were calculated and presented as the total number of events per 1000 person-years among patients with type 1 diabetes or type 2 diabetes. Overall and annual adjusted rates of hyperglycemic crises were calculated using negative binomial estimates adjusted for age (during the year of the event), sex, race/ethnicity, region, and year. Adjusted rates of hyperglycemic crises for subgroups by age, sex, race/ethnicity, annual household income, and insulin use status (for type 2 diabetes only) were calculated using negative binomial estimates adjusted for age (at the index date), sex, race/ethnicity, and region. We tested for differences in the outcome by age, sex, race/ethnicity, income level, insulin use (in type 2 diabetes), and year using Wald tests.

Multivariable negative binomial regression models were also used to examine the association between hyperglycemic crises (dependent variable) and the independent variables outlined above. We evaluated hyperglycemic crises as the total number of events per each person, reporting results as incidence risk ratios (IRRs), 95% CIs, and *P* values. Person-years were used as an exposure to determine the estimated rates of and factors associated with hyperglycemic crises. Secondary analyses examined the outcomes of DKA and of HHS separately. Analyses were conducted using SAS Enterprise Guide, version 7.1 (SAS Institute Inc) and STATA, version 15.1 (StataCorp LLC). A 2-sided *P* < .05 was considered statistically significant.

## Results

### Study Population

The study cohort comprised 20 156 adults with type 1 diabetes (mean [SD] age, 46.6 [16.5] years; 51.2% male; 72.6% White race/ethnicity) and 796 382 adults with type 2 diabetes (mean [SD] age, 65.6 [11.8] years; 50.3% female; 54.4% White race/ethnicity) (Table 1 and Table 2). The mean (SD) durations of observation of patients in the 2 cohorts were 2.4 (1.8) years for type 1 diabetes and 2.6 (1.8) years for type 2 diabetes. Of patients with type 2 diabetes, 20.6% were treated with insulin. A higher proportion of patients with type 2 diabetes than with type 1 diabetes had an annual household income less than $40 000 (30.6% vs 14.9%). The mean (SD) HbA_1c_ level among patients with type 1 diabetes was 8.1% (1.6%), compared with 7.4% (1.6%) among patients with type 2 diabetes (to convert to proportion of total hemoglobin, multiply by 0.01).

**Table 1.** Baseline Sociodemographic, Clinical, and Diabetes Treatment Characteristics of Patients With Type 1 Diabetes

Characteristic	Participants, No. (%)^a^			*P* value
	Total (N = 20 156)	Without hyperglycemic crises (n = 18 883)	With hyperglycemic crises (n = 1273)	
**Sociodemographic variables**				
Age, y				
Mean (SD)	46.6 (16.5)	46.9 (16.4)	43.2 (18.4)	<.001
18-44	9325 (46.3)	8649 (45.8)	676 (53.1)	<.001
45-64	7574 (37.5)	7175 (38.0)	399 (31.3)	
65-74	2367 (11.7)	2237 (11.8)	130 (10.2)	
≥75	890 (4.4)	822 (4.4)	68 (5.3)	
Sex				
Female	9846 (48.8)	9112 (48.3)	734 (57.7)	<.001
Male	10 310 (51.2)	9771 (51.7)	539 (42.3)	
Race/ethnicity				
White	14 630 (72.6)	13 756 (72.8)	874 (68.7)	<.001
Black	1969 (9.8)	1799 (9.5)	170 (13.4)	
Hispanic	1814 (9.0)	1690 (9.0)	124 (9.7)	
Asian	523 (2.6)	492 (2.6)	31 (2.4)	
Other/unknown^b^	1220 (6.0)	1146 (6.1)	74 (5.8)	
Annual household income, $				
<40 000	2995 (14.9)	2730 (14.5)	265 (20.8)	<.001
40 000-74 999	4454 (22.1)	4134 (21.9)	320 (25.1)	
75 000-124 999	5477 (27.2)	5139 (27.2)	338 (26.6)	
125 000-199 999	3283 (16.3)	3133 (16.6)	150 (11.8)	
≥200 000	2468 (12.2)	2368 (12.5)	100 (7.9)	
Unknown	1479 (7.3)	1379 (7.3)	100 (7.9)	
US census region				
Midwest	3855 (19.1)	3581 (19.0)	274 (21.5)	<.001
Northeast	2648 (13.1)	2517 (13.3)	131 (10.3)	
South	9740 (48.3)	9085 (48.1)	655 (51.4)	
West/unknown	3913 (19.4)	3700 (19.6)	213 (16.7)	
Index year				
2014	5151 (25.6)	4791 (25.4)	360 (28.3)	<.001
2015	2628 (13.0)	2448 (13.0)	180 (14.1)	
2016	2977 (14.8)	2780 (14.7)	197 (15.5)	
2017	3036 (15.1)	2834 (15.0)	202 (15.9)	
2018	2931 (14.5)	2754 (14.6)	177 (13.9)	
2019	3433 (17.0)	3276 (17.4)	157 (12.3)	
**Clinical variables**				
Comorbidity				
Hyperglycemic crisis	862 (4.3)	545 (2.9)	317 (24.9)	<.001
Severe hypoglycemia	623 (3.1)	506 (2.7)	117 (9.2)	<.001
Retinopathy	5888 (29.2)	5522 (29.2)	366 (28.8)	.71
Neuropathy	5104 (25.3)	4625 (24.5)	479 (37.6)	<.001
Nephropathy	2956 (14.7)	2671 (14.1)	285 (22.4)	<.001
Cardiovascular disease	2920 (14.5)	2679 (14.2)	241 (18.9)	<.001
Cerebrovascular disease	1038 (5.2)	928 (4.9)	110 (8.6)	<.001
Peripheral vascular disease	1837 (9.1)	1680 (8.9)	157 (12.3)	<.001
Heart failure	692 (3.4)	616 (3.3)	76 (6.0)	<.001
Dementia	173 (0.9)	152 (0.8)	21 (1.6)	.002
Hypertension	9336 (46.3)	8725 (46.2)	611 (48.0)	.21
Depression	2549 (12.6)	2252 (11.9)	297 (23.3)	<.001
COPD	1157 (5.7)	1054 (5.6)	103 (8.1)	<.001
Cancer	832 (4.1)	776 (4.1)	56 (4.4)	.62
Cirrhosis	85 (0.4)	≥74 (0.4)^c^	<11^c^	.47
**Treatment variables**				
Hemoglobin A_1c_ level, %				
Mean (SD)	8.1 (1.6)	8.0 (1.5)	9.4 (2.0)	<.001
≤5.6	354 (1.8)	≥343 (1.8)^c^	<11^c^	<.001
5.7-6.4	1859 (9.2)	1822 (9.6)	37 (2.9)	
6.5-6.9	2444 (12.1)	2378 (12.6)	66 (5.2)	
7.0-7.9	6359 (31.6)	6116 (32.4)	243 (19.1)	
8.0-8.9	4493 (22.3)	4245 (22.5)	248 (19.5)	
9.0-9.9	2387 (11.8)	2164 (11.5)	223 (17.5)	
≥10	2260 (11.2)	1811 (9.6)	449 (35.3)	
Prescription fills for glucose-lowering medication in the 120 d before the index date				
Insulin only	16 805 (83.4)	15 724 (83.3)	1081 (84.9)	<.001
Any noninsulin medication	1829 (9.1)	1749 (9.3)	80 (6.3)	
None	1522 (7.6)	1410 (7.5)	112 (8.8)	

Abbreviation: COPD, chronic obstructive pulmonary disease.

SI conversion factor: To convert hemoglobin A_1c_ to proportion of total hemoglobin, multiply by 0.01.

^a^ Data are overall (total) and by whether patients experienced diabetic ketoacidosis or hyperglycemic hyperosmolar state during the follow-up period.

^b^ Other is a racial/ethnicity choice in the OptumLabs Data Warehouse database.

^c^ Patient counts less than 11 are masked to preserve deidentification. Thus, all numbers lower than 11 are reported as less than 11 and numbers within the same row are presented with less precision to prevent back-calculations of the masked sample.

**Table 2.** Baseline Sociodemographic, Clinical, and Diabetes Treatment Characteristics of Patients With Type 2 Diabetes

Characteristic	Participants, No. (%)^a^			*P* value
	Total (N = 796 382)	Without hyperglycemic crises (n = 790 587)	With hyperglycemic crises (n = 5795)	
**Sociodemographic variables**				
Age, y				
Mean (SD)	65.6 (11.8)	65.7 (11.8)	62.0 (13.7)	<.001
18-44	42 356 (5.3)	41 717 (5.3)	639 (11.0)	<.001
45-64	281 738 (35.4)	279 406 (35.3)	2332 (40.2)	
65-74	286 441 (36.0)	284 676 (36.0)	1765 (30.5)	
≥75	185 847 (23.3)	184 788 (23.4)	1059 (18.3)	
Sex				
Female	400 346 (50.3)	397 272 (50.2)	3074 (53.0)	<.001
Male	396 036 (49.7)	393 315 (49.8)	2721 (47.0)	
Race/ethnicity				
White	433 623 (54.4)	430 499 (54.4)	3124 (53.9)	<.001
Black	141 999 (17.8)	140 476 (17.8)	1523 (26.3)	
Hispanic	133 664 (16.8)	132 901 (16.8)	763 (13.2)	
Asian	40 971 (5.1)	40 836 (5.2)	135 (2.3)	
Other/unknown^b^	46 125 (5.8)	45 875 (5.8)	250 (4.3)	
Annual household income, $				
<40 000	243 621 (30.6)	241 339 (30.5)	2282 (39.4)	<.001
40 000-74 999	222 612 (28.0)	220 975 (28.0)	1637 (28.2)	
75 000-124 999	171 737 (21.6)	170 747 (21.6)	990 (17.1)	
125 000-199 999	64 332 (8.1)	64 057 (8.1)	275 (4.8)	
≥200 000	28 576 (3.6)	28 449 (3.6)	127 (2.2)	
Unknown	65 504 (8.2)	65 020 (8.2)	484 (8.4)	
US census region				
Midwest	144 126 (18.1)	143 046 (18.1)	1080 (18.6)	<.001
Northeast	120 946 (15.2)	120 193 (15.2)	753 (13.0)	
South	446 286 (56.0)	442 879 (56.0)	3407 (58.8)	
West/unknown	85 024 (10.7)	84 469 (10.7)	555 (9.6)	
Index year				
2014	152 519 (19.2)	151 000 (19.1)	1159 (26.2)	<.001
2015	117 351 (14.7)	116 318 (14.7)	1033 (17.8)	
2016	104 538 (13.1)	103 777 (13.1)	761 (13.1)	
2017	137 597 (17.3)	136 555 (17.3)	1042 (18.0)	
2018	142 231 (17.9)	141 433 (17.9)	798 (13.8)	
2019	142 146 (17.8)	141 504 (17.9)	642 (11.1)	
**Clinical variables**				
Comorbidity				
Hyperglycemic crisis	2646 (0.3)	2063 (0.3)	583 (10.1)	<.001
Severe hypoglycemia	6727 (0.8)	6342 (0.8)	385 (6.6)	<.001
Retinopathy	123 148 (15.5)	121 733 (15.4)	1415 (24.4)	<.001
Neuropathy	218 245 (27.4)	215 790 (27.3)	2455 (42.4)	<.001
Nephropathy	186 900 (23.5)	184 944 (23.4)	1956 (33.8)	<.001
Cardiovascular disease	259 985 (32.6)	257 875 (32.6)	2110 (36.4)	<.001
Cerebrovascular disease	92 517 (11.6)	91 584 (11.6)	933 (16.1)	<.001
Peripheral vascular disease	132 101 (16.6)	130 770 (16.5)	1331 (23.0)	<.001
Heart failure	85 846 (10.8)	84 889 (10.7)	957 (16.5)	<.001
Dementia	24 052 (3.0)	23 788 (3.0)	264 (4.6)	<.001
Hypertension	686 124 (86.2)	681 196 (86.2)	4928 (85.0)	.01
Depression	99 487 (12.5)	98 313 (12.4)	1174 (20.3)	<.001
COPD	114 033 (14.3)	112 987 (14.3)	1046 (18.0)	<.001
Cancer	71 797 (9.0)	71 287 (9.0)	510 (8.80)	.57
Cirrhosis	8952 (1.1)	8834 (1.1)	118 (2.0)	<.001
**Treatment variables**				
Hemoglobin A_1c_ level, %				
Mean (SD)	7.4 (1.6)	7.3 (1.6)	9.3 (2.4)	<.001
≤5.6	46 822 (5.9)	46 736 (5.9)	86 (1.5)	<.001
5.7-6.4	217 748 (27.3)	217 308 (27.5)	440 (7.6)	
6.5-6.9	144 631 (18.2)	144 177 (18.2)	454 (7.8)	
7.0-7.9	182 928 (23.0)	181 943 (23.0)	985 (17.0)	
8.0-8.9	90 804 (11.4)	89 817 (11.4)	987 (19.5)	
9.0-9.9	49 029 (6.2)	48 185 (6.1)	844 (14.6)	
≥10	64 420 (8.1)	62 421 (7.9)	1999 (34.5)	
Prescription fills for glucose-lowering medication in the 120 d before the index date				
None	184 079 (23.1)	183 138 (23.2)	941 (16.2)	<.001
Insulin				
Not treated with insulin	632 323 (79.4)	629 706 (79.6)	2617 (45.2)	<.001
Basal only, no bolus	89 977 (11.3)	88 724 (11.2)	1253 (21.6)	
Bolus, with or without basal	74 082 (9.3)	72 157 (9.1)	1925 (33.2)	
Noninsulin glucose-lowering medication				
Sulfonylurea	203 122 (25.5)	201 852 (25.5)	201 852 (25.5)	<.001
Metformin	429 036 (53.9)	426 884 (54.0)	2152 (37.1)	<.001
SGLT2 inhibitor	40 247 (5.0)	39 933 (5.0)	314 (5.4)	.20
GLP-1 receptor agonist	45 950 (5.8)	45 632 (5.8)	318 (5.5)	.35
DPP-4 inhibitor	96 962 (12.2)	96 377 (12.2)	585 (10.1)	<.001
Thiazolidinedione	38 668 (4.9)	38 439 (4.9)	229 (4.0)	.001
Other	7685 (1.0)	7629 (1.0)	56 (1.0)	.99

Abbreviations: COPD, chronic obstructive pulmonary disease; DPP-4, dipeptidyl peptidase 4; GLP-1, glucagon-like peptide 1; SGLT2, sodium-glucose cotransporter 2.

SI conversion factor: To convert hemoglobin A_1c_ to proportion of total hemoglobin, multiply by 0.01.

^a^ Data are overall (total) and by whether patients experienced at least 1 hyperglycemic crisis during the follow-up period.

^b^ Other is a racial/ethnicity choice in the OptumLabs Data Warehouse database.

A total of 1273 patients with type 1 diabetes (6.3%) experienced 2397 episodes of hyperglycemic crises. A total of 2364 events (98.6%) were DKA, with only 33 episodes (1.4%) coded as HHS; thus, no secondary analysis by event type was conducted. The mean (SD) number of hyperglycemic crises among patients with at least 1 event was 1.9 (2.2). Compared with those who did not experience hyperglycemic crises, patients who did were more frequently young (mean [SD] age, 43.2 [18.4] vs 46.9 [16.4] years), Black (170 [13.4%] vs 1799 [9.5%]) or Hispanic (124 [9.7%] vs 1690 [9.0%]) individuals, and female (734 [57.7%] vs 9112 [48.3%]) and had lower income levels (<$40,000: 265 [20.8%] vs 2730 [14.5%]) and higher HbA_1c_ levels (mean [SD], 9.4% [2.0%] vs 8.0% [1.5%]) (Table 1).

Among patients with type 2 diabetes, 5795 patients (0.7%) experienced 8005 hyperglycemic crises. The mean (SD) number of hyperglycemic crises among patients with at least 1 event was 1.4 (1.6). Compared with patients who did not experience hyperglycemic crises, patients who did were younger (mean [SD] age, 62.0 [13.7] vs 65.7 [11.8] years) and more likely to be Black individuals (1523 [26.3%] vs 140 476 [17.8%]) and had lower income levels (<$40 000: 2282 [39.4%] vs 241 339 [30.5%]) and higher HbA_1c_ levels (mean [SD], 9.3% [1.5%] vs 7.3% [1.6%]) (Table 2). When DKA and HHS outcomes were examined separately, 4264 patients (0.5%) experienced only DKA, 1329 (0.2%) experienced only HHS, and 202 (0.02%) experienced both types of events during the study period (eTable 3 in the Supplement). The group who experienced both HHS and DKA was analyzed together with the group with only DKA to ensure that patient deidentification was maintained because the characteristics of the 2 groups were similar. Compared with patients who experienced DKA, patients with HHS were older, were more likely to be Black individuals, had lower income levels, were less likely to have had a prior DKA or HHS event, and had higher prevalence of all comorbidities.

### Incidence Rates of Hyperglycemic Crises

**Figure 1.**
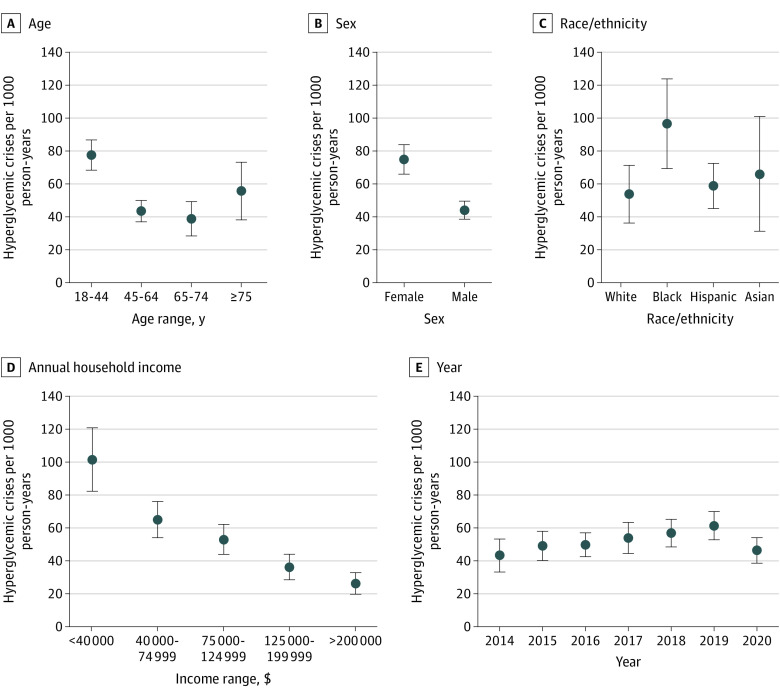
Adjusted Rates of Hyperglycemic Crises Among Adults With Type 1 Diabetes Stratified by Age, Sex, Race/Ethnicity, Annual Household Income, and Year All rates are adjusted for age (age at index for all analyses except for annual rates, which used age at the time of the event), sex, race/ethnicity, US region, and year (for annualized rates). For age, *P* < .001; sex, *P* < .001; race/ethnicity, *P* = .002; annual household income, *P* < .001; and year, *P* = .01.

**Figure 2.**
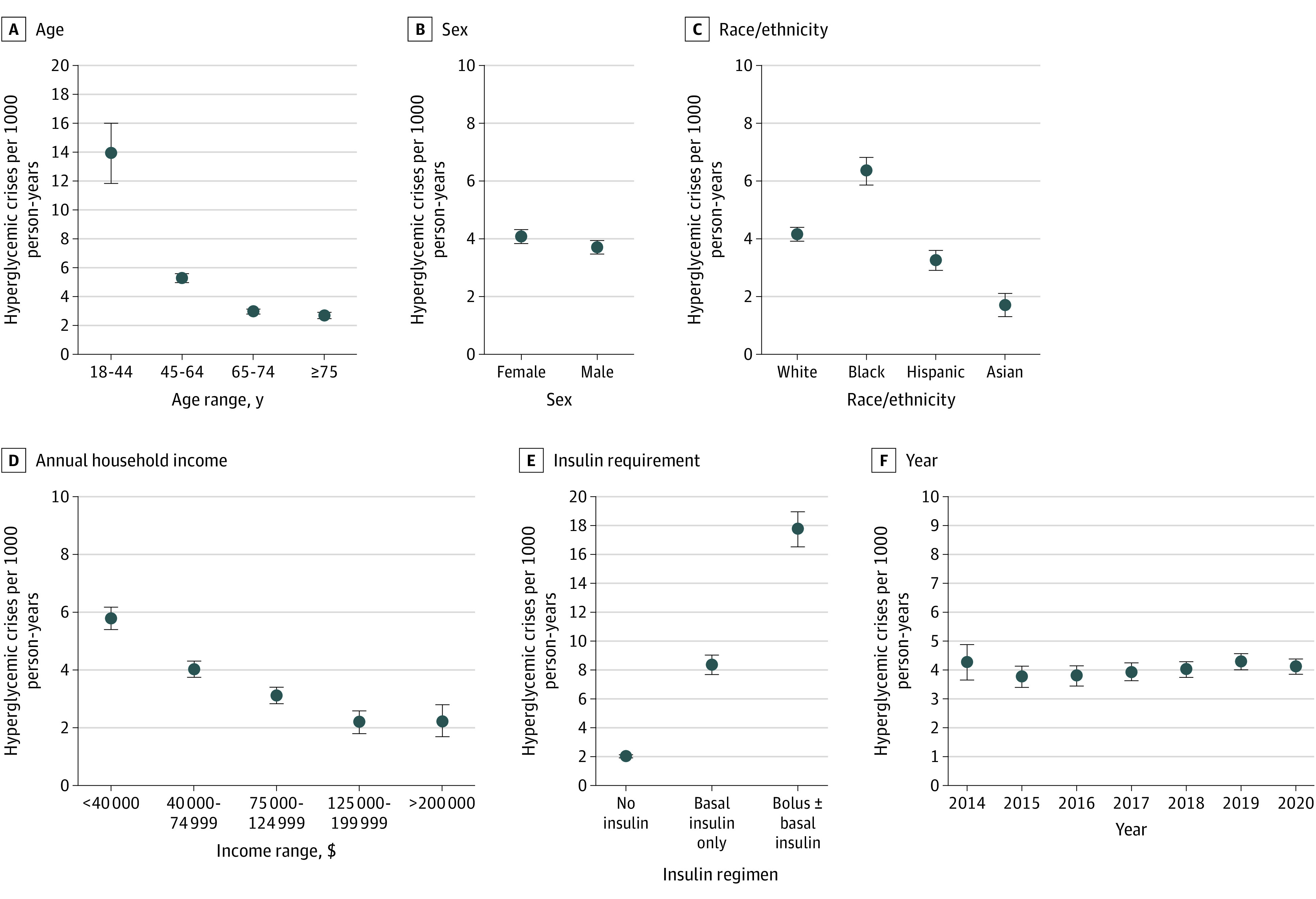
Adjusted Rates of Hyperglycemic Crises Among Adults With Type 2 Diabetes Stratified by Age, Sex, Race/Ethnicity, Annual Household Income, Insulin Therapy, and Year All rates are adjusted for age (age at index for all analyses except for annual rates, which used age at the time of the event), sex, race/ethnicity, US region, and year (for annualized rates). For age, *P* < .001; sex, *P* < .001; race/ethnicity, *P* < .001; annual household income, *P* < .001; insulin requirement, *P* < .001; and year, *P* = .16.

Adjusted overall rates of hyperglycemic crises were 52.69 events per 1000 person-years (95% CI, 48.26-57.12 events per 1000 person-years) among people with type 1 diabetes and 4.04 events per 1000 person-years (95% CI, 3.88-4.21 events per 1000 person-years) among people with type 2 diabetes. Event rates increased between 2014 and 2019 among patients with type 1 diabetes, from 43.30 events (95% CI, 33.37-53.24 events) to 61.36 events (95% CI, 52.90-69.82 events) but then decreased to 46.27 events (95% CI, 38.59-53.95 events) in 2020 (*P* = .01) (Figure 1 and Figure 2 and eTable 4 in the Supplement). In contrast, event rates among patients with type 2 diabetes remained stable throughout the study period. For both patients with type 1 diabetes and those with type 2 diabetes, rates of hyperglycemic crises were highest among younger patients, Black patients, patients with lower income, women, and (for type 2 diabetes) patients requiring insulin therapy (Figure 1 and Figure 2 and eTable 5 and eTable 6 in the Supplement). Indeed, among patients with type 2 diabetes treated with bolus insulin, with or without basal insulin, the adjusted rate of DKA or HHS was 17.73 events per 1000 person-years. Patients with type 1 diabetes whose annual household income was less than $40 000 experienced hyperglycemic crises at the adjusted rate of 101.52 events per 1000 person-years, compared with 26.25 events per 1000 person-years among patients with an income of $200 000 or higher. Similarly, for patients with type 2 diabetes, adjusted rates of hyperglycemic crises were 5.78 events per 1000 person-years for individuals with low income and 2.23 events per 1000 person-years for individuals with high income. Tests of differences by age, sex, racial/ethnic groups, and (for type 2 diabetes) insulin use found significant heterogeneity across all (*P* ≤ .01 for all).

### Factors Associated With Hyperglycemic Crises

For patients with type 1 diabetes and with type 2 diabetes, factors associated with increased risk of hyperglycemic crises were Black race/ethnicity (vs White race/ethnicity: type 1 diabetes incidence risk ratio [IRR], 1.33 [95% CI, 1.01-1.74]; type 2 diabetes IRR, 1.18 [95% CI, 1.09-1.27]) and lower annual household income (≥$200 000 vs <$40 000: type 1 diabetes IRR, 0.61 [95% CI, 0.46-0.81]; type 2 diabetes IRR, 0.69 [95% CI, 0.56-0.86]) (Table 3). Age had a U-shaped association with hyperglycemic crisis risk in patients with type 1 diabetes (compared with patients aged 18-44 years: 45-64 years IRR, 0.72 [95% CI, 0.59-0.87]; 65-74 years IRR, 0.62 [95% CI, 0.47-0.80]; ≥75 years IRR, 0.96 [95% CI, 0.66-1.38]). In type 2 diabetes, risk of hyperglycemic crises decreased progressively with age (45-64 years IRR, 0.57 [95% CI, 0.51-0.63]; 65-74 years IRR, 0.44 [95% CI, .39-0.49]; ≥75 years IRR, 0.41 [95% CI, 0.36-0.47]). In a secondary analysis that considered DKA and HHS separately for patients with type 2 diabetes, age was significantly associated with DKA (≥75 vs 18-44 years: IRR, 0.35; 95% CI, 0.30-0.41) but not with HHS (eTable 7 in the Supplement). Differences by sex were significant only for patients with type 1 diabetes, with men having lower risk than women (IRR, 0.75; 95% CI, 0.65-0.87). Among patients with type 1 diabetes, risk of hyperglycemic crises increased progressively in association with HbA_1c_ levels above 7.0%; at HbA_1c_ levels of 10% or higher, the risk was nearly 8-fold higher than at HbA_1c_ levels from 6.5% to 6.9% (IRR, 7.81; 95% CI, 5.78-10.54). Among patients with type 2 diabetes, risk of hyperglycemic crises increased progressively in association with all HbA_1c_ levels, with an IRR of 0.56 (95% CI, 0.43-0.73) at HbA_1c_ levels of 5.6% or lower and an IRR of 7.06 (95% CI, 6.26-7.96) at HbA_1c_ levels of 10% or higher compared with HbA_1c_ levels from 6.5% to 6.9%. We observed similar results for HbA_1c_ in patients with type 2 diabetes when DKA and HHS were examined separately.

**Table 3.** Factors Associated With Hyperglycemic Crises Among Adults With Type 1 or Type 2 Diabetes, 2014-2020

Factor	Type 1 diabetes		Type 2 diabetes	
	IRR (95% CI)	*P* value	IRR (95% CI)	*P* value
Age, y				
18-44	1 [Reference]	NA	1 [Reference]	NA
45-64	0.72 (0.59-0.87)	.001	0.57 (0.51-0.63)	<.001
65-74	0.62 (0.47-0.80)	<.001	0.44 (0.39-0.49)	<.001
≥75	0.96 (0.66-1.38)	.82	0.41 (0.36-0.47)	<.001
Sex				
Female	1 [Reference]	NA	1 [Reference]	NA
Male	0.75 (0.65-0.87)	<.001	1.00 (0.94-1.07)	.94
Race/ethnicity				
White	1 [Reference]	NA	1 [Reference]	NA
Black	1.33 (1.01-1.74)	.04	1.18 (1.09-1.27)	<.001
Hispanic	0.88 (0.70-1.11)	.28	0.69 (0.62-0.75)	<.001
Asian	1.15 (0.73-1.80)	.55	0.64 (0.51-0.80)	<.001
Other/unknown^a^	1.07 (0.79-1.44)	.68	0.87 (0.73-1.03)	.10
US census region				
Midwest	1 [Reference]	NA	1 [Reference]	NA
Northeast	0.71 (0.56-0.90)	.004	0.92 (0.82-1.03)	.14
South	0.94 (0.78-1.12)	.47	1.01 (0.93-1.09)	.90
West/unknown	0.99 (0.78-1.25)	.90	1.00 (0.88-1.13)	.94
Annual household income, $				
<40 000	1 [Reference]	NA	1 [Reference]	NA
40 000-74 999	0.85 (0.68-1.06)	.15	0.84 (0.78-0.91)	<.001
75 000-124 999	0.92 (0.73-1.17)	.51	0.73 (0.66-0.80)	<.001
125 000-199 999	0.71 (0.54-0.92)	.009	0.57 (0.49-0.66)	<.001
≥200 000	0.61 (0.46-0.81)	.001	0.69 (0.56-0.86)	.001
Unknown	0.98 (0.72-1.35)	.91	1.01 (0.88-1.16)	.87
Index year				
2014	1 [Reference]	NA	1 [Reference]	NA
2015	0.86 (0.69-1.07)	.18	0.92 (0.84-1.01)	.09
2016	1.22 (0.94-1.59)	.13	0.93 (0.83-1.03)	.16
2017	1.08 (0.86-1.37)	.50	0.98 (0.89-1.08)	.66
2018	1.05 (0.85-1.31)	.66	0.99 (0.89-1.09)	.81
2019	1.04 (0.82-1.31)	.76	1.04 (0.93-1.17)	.49
Comorbidity				
Hyperglycemic crisis	7.88 (6.06-9.99)	<.001	17.51 (15.07-20.34)	<.001
Severe hypoglycemia	2.77 (2.15-3.56)	<.001	4.18 (3.58-4.87)	<.001
Retinopathy	0.92 (0.79-1.08)	.31	1.27 (1.18-1.38)	<.001
Nephropathy	1.22 (1.01-1.48)	.04	1.23 (1.14-1.33)	<.001
Neuropathy	1.64 (1.39-1.93)	<.001	1.25 (1.17-1.34)	<.001
Cardiovascular disease	1.22 (0.97-1.53)	.09	0.93 (0.87-1.01)	.08
Cerebrovascular disease	1.04 (0.81-1.34)	.77	1.18 (1.07-1.30)	.001
Peripheral vascular disease	1.18 (0.92-1.51)	.20	1.09 (1.00-1.18)	.05
Heart failure	0.88 (0.64-1.22)	.45	1.14 (1.03-1.25)	.01
Dementia	1.50 (0.89-2.52)	.13	1.49 (1.27-1.75)	<.001
Hypertension	0.93 (0.79-1.10)	.39	0.83 (0.76-0.91)	<.001
Depression	1.62 (1.37-1.92)	<.001	1.46 (1.34-1.59)	<.001
COPD	1.03 (0.79-1.34)	.85	1.11 (1.02-1.21)	.02
Cancer	1.09 (0.77-1.53)	.63	1.15 (1.03-1.28)	.01
Cirrhosis	1.09 (0.51-2.37)	.82	1.72 (1.36-2.18)	<.001
Hemoglobin A_1c,_ %				
≤5.6	0.56 (0.27-1.14)	.11	0.56 (0.43-0.73)	<.001
5.7-6.4	0.99 (0.58-1.68)	.96	0.67 (0.58-0.77)	<.001
6.5-6.9	1 [Reference]	NA	1 [Reference]	NA
7.0-7.9	1.63 (1.20-2.22)	.002	1.48 (1.31-1.67)	<.001
8.0-8.9	2.11 (1.55-2.86)	<.001	2.61 (2.30-2.96)	<.001
9.0-9.9	3.57 (2.61-4.89)	<.001	3.72 (3.25-4.27)	<.001
≥10	7.81 (5.78-10.54)	<.001	7.06 (6.26-7.96)	<.001
Type 1 diabetes management				
Insulin only	1 [Reference]	NA	NA	NA
No medications	1.05 (0.80-1.38)	.72	NA	NA
Any noninsulin medication	0.65 (0.49-0.87)	.003	NA	NA
Type 2 diabetes management				
No medications	NA	NA	1.10 (0.98-1.23)	.09
Insulin use				
Bolus with or without basal	NA	NA	1 [Reference]	NA
Basal only	NA	NA	0.69 (0.63-0.75)	<.001
Not treated with insulin	NA	NA	0.36 (0.33-0.40)	<.001
Noninsulin medication				
Metformin	NA	NA	0.72 (0.67-0.78)	<.001
Sulfonylurea	NA	NA	0.90 (0.84-0.98)	.01
SGLT2 inhibitor	NA	NA	1.30 (1.14-1.49)	<.001
GLP-1 receptor agonist	NA	NA	0.77 (0.67-0.87)	<.001
DPP-4 inhibitor	NA	NA	0.87 (0.79-0.96)	.006
Thiazolidinedione	NA	NA	1.12 (0.96-1.31)	.15
Other	NA	NA	0.86 (0.64-1.15)	.30

Abbreviations: COPD, chronic obstructive pulmonary disease; DPP-4, dipeptidyl peptidase 4; GLP-1, glucagon-like peptide 1; IRR, incidence risk ratio; NA, not applicable; SGLT2, sodium-glucose cotransporter 2.

SI conversion factor: To convert hemoglobin A_1c_ to proportion of total hemoglobin, multiply by 0.01.

^a^ Other is a racial/ethnicity choice in the OptumLabs Data Warehouse database.

Patients who experienced hyperglycemic crises or severe hypoglycemia in the previous year were significantly more likely to experience recurrent DKA or HHS (Table 3). Among patients with type 1 diabetes, both prior hyperglycemic crises (IRR, 7.88; 95% CI, 6.06-9.99) and prior severe hypoglycemia (IRR, 2.77; 95% CI, 2.15-3.56) were associated with increased risk of experiencing hyperglycemic crises. Similarly, among patients with type 2 diabetes, prior hyperglycemic crises (IRR, 17.51; 95% CI, 15.07-20.34) and prior severe hypoglycemia (IRR, 4.18; 95% CI, 3.58-4.87) were associated with increased risk of hyperglycemic crises. When DKA and HHS were examined separately, history of hyperglycemic crises had a greater association with DKA (IRR, 20.32; 95% CI, 17.31-23.86) than HHS (IRR, 7.18; 95% CI, 5.68-9.06) (eTable 7 in the Supplement).

Compared with patients without the following health conditions, patients with depression (type 1 diabetes IRR, 1.62 [95% CI, 1.37-1.92]; type 2 diabetes IRR, 1.46 [95% CI, 1.34-1.59]), neuropathy (type 1 diabetes IRR, 1.64 [95% CI, 1.39-1.93]; type 2 diabetes IRR, 1.25 [95% CI, 1.17-1.34]), and nephropathy (type 1 diabetes IRR, 1.22 [95% CI, 1.01-1.48]; type 2 diabetes IRR, 1.23 [95% CI, 1.14-1.33]) had an increased risk of hyperglycemic crises (Table 3). For type 2 diabetes, additional risk was associated with the presence of retinopathy, cerebrovascular disease, heart failure, dementia, chronic obstructive pulmonary disease, cirrhosis, or cancer.

Patients with type 2 diabetes who required basal insulin therapy (IRR, 0.69; 95% CI, 0.63-0.75) or no insulin therapy (IRR, 0.36; 95% CI, 0.33-0.40) had a lower risk of hyperglycemic crises compared with those treated with bolus, with or without basal, insulin therapy. Patients treated with sodium-glucose cotransporter 2 inhibitors (IRR, 1.30; 95% CI, 1.14-1.49) had an increased risk of hyperglycemic crises compared with patients not treated with these medications (Table 3). Therapy with a sodium-glucose cotransporter 2 inhibitor was specifically associated with an increased risk of DKA (IRR, 1.47; 95% CI, 1.27-1.70) but with a decreased risk of HHS (IRR, 0.65; 95% CI, 0.46-0.93) (eTable 7 in the Supplement).

## Discussion

In this nationwide cohort study of insured adults with diabetes, adjusted rates of hyperglycemic crises were 52.69 events per 1000 person-years among people with type 1 diabetes, 4.04 events per 1000 person-years among people with type 2 diabetes, and 17.73 events per 1000 person-years among people with type 2 diabetes requiring intensive insulin therapy. In both patients with type 1 diabetes and those with type 2 diabetes, rates of hyperglycemic crises were significantly higher among younger adults, Black patients, individuals with lower income, patients with elevated HbA_1c_ levels, and patients with prior hyperglycemic crises or severe hypoglycemia, suggesting the need for timely patient identification, engagement, and treatment optimization to improve glycemic control and prevent these dangerous events.

We found that the rates of hyperglycemic crises increased over time among patients with type 1 diabetes, from 43.30 per 1000 person-years in 2014 to 61.36 per 1000 person-years in 2019, consistent with previously described trends through 2015^16^ and recent population-level data showing an overall worsening of glycemic control among patients with diabetes in the US.^23,24^ There was no comparable temporal change in hyperglycemic crises rates among patients with type 2 diabetes. This persistent rise in rates of hyperglycemic crises among patients with type 1 diabetes underscores the urgency of improving glycemic control and diabetes management in this population. Why ED visits and hospitalizations for hyperglycemic crises among patients with type 1 diabetes declined in 2020 in the context of the COVID-19 pandemic is unknown. Although there may have been a delay in adjudication of claims, with not all 2020 claims finalized at the time of our final analysis (June 2021), this delay alone likely did not account for the observed decrease in hyperglycemic crises–associated ED visits or hospitalizations to 46.27 per 1000 person-years in 2020 (particularly as a similar decrease was not observed for patients with type 2 diabetes). Patients may have sought to avoid the ED or hospital to minimize COVID-19 exposure or because EDs and hospitals were overwhelmed by caring for patients with COVID-19. Alternatively, patients may have observed improvements in diabetes management in the context of evolving policy changes regarding insulin access and affordability. Further exploration will be needed to examine diabetes management in 2020, particularly among patients with type 1 diabetes, and to assess for changes in insulin use and adherence, glycemic control, and diabetes-related mortality.

In the present study, incidence and risk of hyperglycemic crises was disproportionately higher for patients with lower income, a finding supporting the need for policies and systems to ensure improved access to affordable glucose-lowering therapies.^25,26^ Building on earlier work in different populations,^27,28^ we found that for individuals with type 1 diabetes, the adjusted rate of hyperglycemic crises was 101.52 events per 1000 person-years among patients with low income (<$40 000) compared with 26.25 events per 1000 person-years among patients with high income (≥$200 000). Income-based disparities in hyperglycemic crisis risk were also detected among patients with type 2 diabetes: 5.78 vs 2.23 per 1000 person-years, respectively. The association of an inability to afford insulin with increased risk of DKA has been observed for decades.^29^ Increasing costs of insulin^26,30,31^ are associated with an increased prevalence of undertreatment of patients with lower incomes^32^ and insulin rationing,^33^ which may lead to severe and uncontrolled hyperglycemia as a result of inadequate access to the medication that all patients with type 1 diabetes and some with insulin-requiring type 2 diabetes need to live. Although rates of hyperglycemic emergencies may be higher among uninsured patients owing to poor access to medical care, healthy food, and housing,^16,34^ many people with employer-sponsored private health plans have high deductibles and out-of-pocket cost-sharing expenses,^35^ which may be associated with financial distress^36^ and greater likelihood of forgoing necessary medical care.^37^ Young patients may be particularly susceptible to financial instability and underinsurance,^34,38^ which may be associated with poor glycemic control,^21,34,39,40^ inadequate ambulatory care, and ultimately, higher rates of hyperglycemic crises, like those observed in our study. Thus, multidisciplinary teams should anticipate these barriers to care, develop care plans that are affordable and accessible, and help patients navigate available support programs.

We believe that greater attention should be given to addressing racial/ethnic disparities in diabetes management. Black patients with type 1 diabetes or with type 2 diabetes had higher risks of hyperglycemic crises than individuals in the other racial/ethnic groups included in the study. This disparity persisted after adjustment for key socioeconomic, clinical, and treatment-related factors, suggesting that additional intrinsic and extrinsic factors are associated with hyperglycemic crises among Black patients. Black patients may be more likely to experience DKA in the context of ketosis-prone type 2 diabetes, which is more prevalent among Black patients.^41,42,43^ In addition to biological risk factors, increased DKA and HHS risk among Black patients may be associated with unmeasured social determinants of health and manifestations of structural racism both within and outside health care.^44^ Black patients with type 2 diabetes are more likely than White patients to be undertreated, and undertreated patients are more likely to experience hyperglycemic crises than patients who were treated appropriately.^32^ Black patients are also more likely to be cared for by clinicians^45^ and health systems^46^ that deliver lower-quality care or have fewer resources available to optimally care for their patients,^45^ which may contribute to worse health outcomes. Furthermore, Black patients are more likely to reside in less walkable neighborhoods^47^ with fewer healthy food options,^48,49,50^ which may be associated with worse glycemic control. Thus, additional research is needed to understand the multiplicity of factors contributing to the undertreatment of Black patients with diabetes, barriers to managing their diabetes, and their increased risk of hyperglycemic crises.

Poorly controlled diabetes was another factor associated with hyperglycemic crises. For patients with type 1 diabetes, risk of experiencing a hyperglycemic crisis increased when the HbA_1c_ level exceeded 7%, and the IRR was 7.81 (95% CI, 5.78-10.54) for HbA_1c_ levels of 10% or higher compared with HbA_1c_ levels of 6.5% to 6.9%. For patients with type 2 diabetes, the risk increased continuously for all HbA_1c_ levels above 5.6%, and the IRR was 7.06 (95% CI, 6.26-7.96) for HbA_1c_ levels of 10% or higher. This association of HbA_1c_ level with increased risk of hyperglycemic emergencies overlooks the importance of real-time glycemic variability that would be captured by self-monitoring or continuous glucose monitoring. The importance of glycemic variability is underscored by the finding that severe hypoglycemia was associated with a 3- to 4-fold increase in the risk of experiencing a hyperglycemic crisis. Because patients with high HbA_1c_ levels also frequently experience severe hypoglycemia,^11^ an elevated HbA_1c_ level is a signal of susceptibility to both hyperglycemic and hypoglycemic crises.

### Strengths and Limitations

This study is strengthened by the ability to examine patient- and treatment-level factors associated with hyperglycemic crises at the population level using longitudinal analysis^16^ and is not limited to a single health care system.^16^ The present study also provides, to our knowledge, the most contemporary epidemiological data on DKA and HHS through the end of 2020, including during the COVID-19 pandemic in the US.

This study also has limitations. First, this observational analysis was designed to examine the epidemiological features of and factors associated with hyperglycemic crises, not to establish a causal relationship between any factor(s) and these events. The classification scheme for type of diabetes and the adjudication of events as being DKA or HHS may not be accurate because both determinations were limited by data available in the claims database. Because all patients had established diabetes, we did not capture instances of newly diagnosed diabetes in patients presenting with DKA (particularly of interest in the context of COVID-19) or assess the impact of lapsed insurance. Our data also did not allow us to examine the factors leading up to and precipitating hyperglycemic events, such as medication nonadherence or acute illness.^2,34^ However, our objectives were to characterize the frequency of hyperglycemic crises among adults with type 1 diabetes or with type 2 diabetes in the US and to identify patient- and treatment-related factors associated with these events. These epidemiologic data are necessary to inform targeted interventions at multiple levels to prevent hyperglycemic crises among individuals at highest risk of these conditions.

## Conclusions

In this cohort study, younger age, Black race/ethnicity, low income, and poor glycemic control were associated with an increased risk of hyperglycemic crises. The findings suggest that efforts are needed to facilitate engagement of these groups with the health care system, assess and address potential barriers—financial, logistical, psychosocial, or medical—to optimal control of patients’ blood glucose levels, and provide referral for diabetes self-management education and support.^15,51^ Health care delivery systems should incorporate educational, clinical, and social support systems into clinical practice, and payers should consider expanding reimbursement for self-management education and social services as well as more comprehensive coverage for glucose-lowering medications, insulin, and glucose-monitoring technologies.

## Notes

Supplement. **eTable 1.** Code Sets for Included Health Conditions **eTable 2.** Classification Scheme for Glucose-Lowering Medications **eTable 3.** Patients With Type 2 Diabetes **eTable 4.** Crude and Adjusted Rates of Hyperglycemic Crises Among Patients With Type 1 and Type 2 Diabetes, 2014-2020 **eTable 5.** Crude and Adjusted Rates of Hyperglycemic Crises Among Patients With Type 1 Diabetes by Prespecified Subgroup, 2014-2020 **eTable 6.** Crude and Adjusted Rates of Hyperglycemic Crises Among Patients With Type 2 Diabetes by Prespecified Subgroup, 2014-2020 **eTable 7.** Risk Factors for DKA and HHS (Examined as Independent Outcomes) Among Adults With Type 2 Diabetes, 2014-2020
