# Supplementary material for: Sociodemographic, Clinical, and Treatment-Related Factors Associated With Hyperglycemic Crises Among Adults With Type 1 or Type 2 Diabetes in the US From 2014 to 2020
Source: JAMA Netw Open. 2021 Sep 1;4(9):e2123471. doi: 10.1001/jamanetworkopen.2021.23471 (PMC8411297; doi:10.1001/jamanetworkopen.2021.23471)
Supplement: Supplement. — eTable 1. Code Sets for Included Health Conditions eTable 2. Classification Scheme for Glucose-Lowering Medications eTable 3. Patients With Type 2 Diabetes eTable 4. Crude and Adjusted Rates of Hyperglycemic Crises Among Patients With Type 1 and Type 2 Diabetes, 2014-2020 eTable 5. Crude and Adjusted Rates of Hyperglycemic Crises Among Patients With Type 1 Diabetes by Prespecified Subgroup, 2014-2020 eTable 6. Crude and Adjusted Rates of Hyperglycemic Crises Among Patients With Type 2 Diabetes by Prespecified Subgroup, 2014-2020 eTable 7. Risk Factors for DKA and HHS (Examined as Independent Outcomes) Among Adults With Type 2 Diabetes, 2014-2020 [file jamanetwopen-e2123471-s001.pdf]

Supplementary Online Content

McCoy RG, Galindo RJ, Swarna KS, et al. Sociodemographic, clinical, and treatment-related factors associated with hyperglycemic crises among adults with type 1 or type 2 diabetes in the US from 2014 to 2020. *JAMA Netw Open*. 2021;4(9):e2123471. doi:10.1001/jamanetworkopen.2021.23471

eTable 1. Code Sets for Included Health Conditions
eTable 2. Classification Scheme for Glucose-Lowering Medications
eTable 3. Patients With Type 2 Diabetes
eTable 4. Crude and Adjusted Rates of Hyperglycemic Crises Among Patients With Type 1 and Type 2 Diabetes, 2014-2020
eTable 5. Crude and Adjusted Rates of Hyperglycemic Crises Among Patients With Type 1 Diabetes by Prespecified Subgroup, 2014-2020
eTable 6. Crude and Adjusted Rates of Hyperglycemic Crises Among Patients With Type 2 Diabetes by Prespecified Subgroup, 2014-2020
eTable 7. Risk Factors for DKA and HHS (Examined as Independent Outcomes) Among Adults With Type 2 Diabetes, 2014-2020

This supplementary material has been provided by the authors to give readers additional information about their work.

© 2021 McCoy RG et al. *JAMA Network Open*.

eTable 1. Code Sets for Included Health Conditions. All conditions were ascertained during the baseline 12 months preceding the index date for cohort entry

Comorbidity	ICD-9 Codes	ICD-10 codes	Included code types
**Cancer (except non-melanoma skin cancer)**	14x.xx, 15x.xx, 160.x-165.x, 170.x-172.x, 174.x-176.x, 179-189.x, 190.x-195.x, 199.xx-208.xx (except 203.x1, 204.x1, 205.x1, 206.x1, 207.x1, 208.x1)	C00.x-C14.x, C15.x-C26.x, C3x.xx, C40.xx-C41.x, C43.x, C4A.xx, C45.x-C49.xx, C50.xxx, C51.x-C58, C60.x-C63.x, C64.x-C68.x, C69.xx-C72.x, C73-C76.x, C7A.xx, C80.xx-C96.x (except C90.x1, C91.x1, C92.x1, C93.x1, C94.x1, C95.x1)	All claims<br>Codes in all positions
**Cardiovascular disease**	410.x, 411.x, 412, 413.x, 414.x, 427.1, 427.3x, 427.4x, 427.5, 429.2, 440.x, 441.x	I20.x, I21.x, I22.x, I23.x, I24.x, I25.x, I46.x, I47.x, I48.x, I49.x, I70.x, I71.x	All claims<br>Codes in all positions
**Cerebrovascular disease**	430, 431, 432.x, 433.xx, 434.xx 435.x, 436, 437.x, 438.xx, V12.54	G45.0, G45.1, G45.2, G45.8, G45.9, G46.x, I60.xx, I61.x (except I61.0), I62.xx, I63.xxx, I65.xx, I66.xx, I67.8x (except I67.83, I67.84), I67.9, I69.xxx, Z86.73	All claims<br>Codes in all positions
**Chronic obstructive pulmonary disease**	490, 491.xx, 492.x, 494.x, 496	J40, J41.x, J42, J43.x, J44.x, J47.x	All claims<br>Codes in all positions
**Cirrhosis**	571.2, 571.5, 571.6	K70.3x, K74.3, K74.4, K74.5, K74.6x	All claims<br>Codes in all positions
**Dementia**	046.1x, 290.1x, 290.2x, 290.3, 290.4x, 291.2, 292.82, 294.1x, 294.2x, 331.0, 331.1x, 331.2, 331.6, 331.82, 331.89	A81.0x, F01.5x, F02.8x, F03.9x, F10.27, F10.97, F13.27, F13.97, F18.97, F19.17, F19.27, F19.97, G30.x, G31.0x, G31.1, G31.83, G31.85	All claims<br>Codes in all positions
**Depression**	290.13, 290.21, 290.43, 296.2x, 296.3x, 296.82, 298.0, 301.12, 309.0, 309.1, 309.28, 311	F01.51, F32.x (except F32.81), F33.x, F34.8x, F43.21, F43.23	All claims<br>Codes in all positions
**DKA**	250.10, 250.11, 250.12, 250.13	E10.10, E10.11, E11.10, E11.11, E13.10, E13.11	Hospital and ED claims<br>Codes in 1st position
**HHS**	250.20, 250.21, 250.22, 250.23	E11.00, E11.01, E13.00, E13.01	Hospital and ED claims<br>Codes in 1st position
**Heart failure**	398.91, 402.01, 402.11, 402.91, 404.01, 404.03, 404.11, 404.13, 404.91, 404.93, 428.xx	I09.81, I11.0, I13.0, I13.2, I50.xx	All claims<br>Codes in all positions
**Hypoglycemia**	251.0, 251.1, 251.2, 962.3, 250.8x (for 250.8x: if no concurrent 259.8, 272.7, 681.xx, 682.xx, 686.9x,	E10.641, E10.649, E11.641, E11.649, E13.641, E13.649, E16.0, E16.1, E16.2, T38.3X1A, T38.3X1D, T38.3X1S,	Hospital and ED claims<br>Codes in 1st position

© 2021 McCoy RG et al. *JAMA Network Open*.

Comorbidity	ICD-9 Codes	ICD-10 codes	Included code types
	707.1x-707.2x, 707.8, 707.9, 709.3, 730.0x-730.2x, 731.8)	T38.3X2A, T38.3X2D, T38.3X2S, T38.3X3A, T38.3X3D, T38.3X3S, T38.3X4A, T383X4D, T38.3X4S, T38.3X5A, T383X5D, T38.3X5S	
**Hypertension**	401.x, 402.x, 403.x, 404.x, 405.x	I11.x, I12.x, I13.x, I15.x, I16.x, I10	All claims Codes in all positions
**Nephropathy**	593.9, 586, 250.4x, 249.4x, 580.x, 581.x, 582.x, 583.x, 585.x	E08.21, E08.22, E08.29, E09.21, E09.22, E09.29, E10.21, E10.22, E10.29, E11.21, E11.22, E11.29, E13.21, E13.22, E13.29, N19, N00.x, N03.x, N04.x, N05.x, N18.x	All claims Codes in all positions
**Neuropathy**	357.2, 337.1, 356.9, 358.1, 458.0, 536.3, 564.5, 596.54, 713.5, 951.0, 951.1, 951.3, 250.6x, 249.6x, 337.0x, 354.x, 355.x	G90.09, G90.8, G90.9, G99.0, G60.9, G73.3, G90.01, I95.1, K31.84, K59.1, N31.9, E08.4x, E09.4x, E10.4x, E11.4x, E13.4x, G56.x, G57.x, H49.x, M14.6x, S04.x	All claims Codes in all positions
**Peripheral vascular disease**	442.3, 440.21, 443.81, 443.9, 892.1, 040.0, 444.22, 785.4, 250.7x, 249.7, 707.1x	E08.51, E09.51, E10.51, E11.51, E13.51, E08.59, E09.59, E10.59, E11.59, E13.59, E08.621, E09.621, E10.621, E11.621, E13.621, I72.4, I73.89, I73.9, A48.0, I74.3, I96, E08.52, E09.52, E10.52, E11.52, E13.52, I70.21x, S91.3x, L97.x	All claims Codes in all positions
**Retinopathy**	362.01, 362.03, 362.04, 362.05, 362.06, 362.07, 362.53, 362.81, 362.82, 362.83, 362.02, 379.23, 250.5x, 249.5x, 362.1x, 361.x, 369.x	H35.9, E08.3x, E09.3x, E10.3x, E11.3x, E13.3x, H35.0x, H35.35x, H35.6x, H35.8x, H33.x, H54.x, H43.1x	All claims Codes in all positions

Abbreviations: DKA, diabetic ketoacidosis; ED, emergency department; HHS, hyperglycemic hyperosmolar state.

© 2021 McCoy RG et al. *JAMA Network Open*.

eTable 2. Classification Scheme for Glucose-Lowering Medications

Medication class	Included agents
**Insulin: Basal**	NPH/isophane<br>Determir<br>Glargine<br>Degludec
**Insulin: Bolus**	Regular<br>Aspart<br>Lispro<br>Glulisine<br>Inhaled powder insulin
**Sulfonylureas**	Glimepiride<br>Glipizide<br>Glyburide
**Biguanides**	Metformin
**GLP-1 receptor agonists**	Exenatide<br>Liraglutide<br>Albiglutide<br>Dulaglutide<br>Lixisenatide<br>Semaglutide
**SGLT2 inhibitors**	Canagliflozin<br>Empagliflozin<br>Dapagliflozin<br>Ertugliflozin
**DPP-4 inhibitors**	Sitagliptin<br>Saxagliptin<br>Linagliptin<br>Alogliptin
**Glitazones**	Pioglitazone<br>Rosiglitazone
**Glinides**	Nateglinide<br>Repaglinide
**Amylin analogs**	Pramlintide
**α-Glucosidase inhibitors**	Acarbose<br>Miglitol

© 2021 McCoy RG et al. *JAMA Network Open*.

eTable 3. Patients With Type 2 Diabetes. Baseline sociodemographic, clinical, and diabetes treatment characteristics of included adults with type 2 diabetes, overall and subset by whether they experienced at least one episode of hyperglycemic crisis during the follow-up period. [a] There were 4,264 patients who experienced at least one episode of DKA and 202 patients who experienced at least one episode of DKA and at least one episode of HHS during the observation period. Because of the small sample size of patients who experienced both DKA and HHS events, and our inability to present sample sizes smaller than 11 (as would be the case in many cells for this small group), the group with both types of events was combined with the group experiencing DKA only.

	**Total**	**No DKA/HHS**	**DKA ± HHS [a]**	**HHS**	**p-value**
**Number of patients**	796,382	790,587	4,466	1,329	
**Sociodemographic variables**					
**Age, years, mean (SD)**	65.63 (11.79)	65.66 (11.78)	60.54 (13.95)	66.81 (11.53)	<0.001
**Age, N (%)**					<0.001
18-44 years	42,356 (5.32%)	41,717 (5.28%)	585 (13.10%)	54 (4.06%)	
45-64 years	281,738 (35.38%)	279,406 (35.34%)	1,902 (42.59%)	430 (32.36%)	
65-74 years	286,441 (35.97%)	284,676 (36.01%)	1,275 (28.55%)	490 (36.87%)	
≥75 years	185,847 (23.34%)	184,788 (23.37%)	704 (15.76%)	355 (26.71%)	
**Sex, N (%)**					
Female	400,346 (50.27%)	397,272 (50.25%)	2,380 (53.29%)	694 (52.22%)	
Male	396,036 (49.73%)	393,315 (49.75%)	2,086 (46.71%)	635 (47.78%)	
**Race/ethnicity, N (%)**					<0.001
White	433,623 (54.45%)	430,499 (54.45%)	2,502 (56.02%)	622 (46.80%)	
Black	141,999 (17.83%)	140,476 (17.77%)	1,088 (24.36%)	435 (32.73%)	
Hispanic	133,664 (16.78%)	132,901 (16.81%)	592 (13.26%)	171 (12.87%)	
Asian	40,971 (5.14%)	40,836 (5.17%)	93 (2.08%)	42 (3.16%)	
Other/unknown	46,125 (5.79%)	45,875 (5.80%)	191 (4.28%)	59 (4.44%)	
**Annual household income, N (%)**					<0.001
<$40,000	243,621 (30.59%)	241,339 (30.53%)	1,647 (36.88%)	635 (47.78%)	
$40,000-$74,999	222,612 (27.95%)	220,975 (27.95%)	1,284 (28.75%)	353 (26.56%)	
$75,000-$124,999	171,737 (21.56%)	170,747 (21.60%)	815 (18.25%)	175 (13.17%)	
$125,000-$199,999	64,332 (8.08%)	64,057 (8.10%)	239 (5.35%)	36 (2.71%)	
≥$200,000	28,576 (3.59%)	28,449 (3.60%)	108 (2.42%)	19 (1.43%)	
Unknown	65,504 (8.23%)	65,020 (8.22%)	373 (8.35%)	111 (8.35%)	
**Index year, N (%)**					<0.001
2014	152,519 (19.15%)	151,000 (19.10%)	1,193 (26.71%)	326 (24.53%)	
2015	117,351 (14.74%)	116,318 (14.71%)	788 (17.64%)	245 (18.43%)	
2016	104,538 (13.13%)	103,777 (13.13%)	603 (13.50%)	158 (11.89%)	
2017	137,597 (17.28%)	136,555 (17.27%)	767 (17.17%)	275 (20.69%)	
2018	142,231 (17.86%)	141,433 (17.89%)	619 (13.86%)	179 (13.47%)	
2019	142,146 (17.85%)	141,504 (17.90%)	496 (11.11%)	146 (10.99%)	

© 2021 McCoy RG et al. *JAMA Network Open*.

	Total	No DKA/HHS	DKA ± HHS [a]	HHS	p-value
**U.S. census region, N (%)**					<0.001
Midwest	144,126 (18.10%)	143,046 (18.09%)	829 (18.56%)	251 (18.89%)	
Northeast	120,946 (15.19%)	120,193 (15.20%)	538 (12.05%)	215 (16.18%)	
South	446,286 (56.04%)	442,879 (56.02%)	2,643 (59.18%)	764 (57.49%)	
West/unknown	85,024 (10.68%)	84,469 (10.68%)	456 (10.21%)	99 (7.45%)	
**Clinical variables**					
**Comorbidities, N (%)**					
Hyperglycemic crisis	2,646 (0.33%)	2,063 (0.26%)	510 (11.42%)	73 (5.49%)	<0.001
Severe hypoglycemia	6,727 (0.84%)	6,342 (0.80%)	305 (6.83%)	80 (6.02%)	<0.001
Retinopathy	123,148 (15.46%)	121,733 (15.40%)	1,093 (24.47%)	322 (24.23%)	<0.001
Neuropathy	218,245 (27.40%)	215,790 (27.29%)	1,883 (42.16%)	572 (43.04%)	<0.001
Nephropathy	186,900 (23.47%)	184,944 (23.39%)	1,382 (30.94%)	574 (43.19%)	<0.001
Cardiovascular disease	259,985 (32.65%)	257,875 (32.62%)	1,527 (34.19%)	583 (43.87%)	<0.001
Cerebrovascular disease	92,517 (11.62%)	91,584 (11.58%)	643 (14.40%)	290 (21.82%)	<0.001
Peripheral vascular disease	132,101 (16.59%)	130,770 (16.54%)	960 (21.50%)	371 (27.92%)	<0.001
Heart failure	85,846 (10.78%)	84,889 (10.74%)	636 (14.24%)	321 (24.15%)	<0.001
Dementia	24,052 (3.02%)	23,788 (3.01%)	176 (3.94%)	88 (6.62%)	<0.001
Hypertension	686,124 (86.16%)	681,196 (86.16%)	3,680 (82.40%)	1,248 (93.91%)	<0.001
Depression	99,487 (12.49%)	98,313 (12.44%)	918 (20.56%)	256 (19.26%)	<0.001
COPD	114,033 (14.32%)	112,987 (14.29%)	727 (16.28%)	319 (24.00%)	<0.001
Cancer	71,797 (9.02%)	71,287 (9.02%)	365 (8.17%)	145 (10.91%)	0.008
Cirrhosis	8,952 (1.12%)	8,834 (1.12%)	77 (1.72%)	41 (3.09%)	<0.001
**Treatment variables**					
**Hemoglobin A_{1c}, %, mean (SD)**	7.36 (1.63)	7.34 (1.61)	9.37 (2.33)	8.91 (2.42)	<0.001
**Hemoglobin A_{1c}, N (%)**					<0.001
≤5.6%	46,822 (5.88%)	46,736 (5.91%)	57 (1.28%)	29 (2.18%)	
5.7% – 6.4%	217,748 (27.34%)	217,308 (27.49%)	288 (6.45%)	152 (11.44%)	
6.5% – 6.9%	144,631 (18.16%)	144,177 (18.24%)	317 (7.10%)	137 (10.31%)	
7.0% – 7.9%	182,928 (22.97%)	181,943 (23.01%)	729 (16.32%)	256 (19.26%)	
8.0% – 8.9%	90,804 (11.40%)	89,817 (11.36%)	800 (17.91%)	187 (14.07%)	
9.0% – 9.9%	49,029 (6.16%)	48,185 (6.09%)	677 (15.16%)	167 (12.57%)	
≥10%	64,420 (8.09%)	62,421 (7.90%)	1,598 (35.78%)	401 (30.17%)	
**Fills for glucose-lowering medication in the 120 days prior to index date**					
**No fills**	184,079 (23.11%)	183,138 (23.16%)	709 (15.88%)	232 (17.46%)	
**Insulin fills, N (%)**					<0.001
Not treated with insulin	632,323 (79.40%)	629,706 (79.65%)	1,924 (43.08%)	693 (52.14%)	
Basal only (no bolus)	89,977 (11.30%)	88,724 (11.22%)	958 (21.45%)	295 (22.20%)	

© 2021 McCoy RG et al. *JAMA Network Open*.

	Total	No DKA/HHS	DKA ± HHS [a]	HHS	p-value
Bolus (± basal)	74,082 (9.30%)	72,157 (9.13%)	1,584 (35.47%)	341 (25.66%)	
**Non-insulin glucose-lowering medications, N (%)**					
Sulfonylurea	203,122 (25.51%)	201,852 (25.53%)	930 (20.82%)	340 (25.58%)	<0.001
Metformin	429,036 (53.87%)	426,884 (54.00%)	1,641 (36.74%)	511 (38.45%)	<0.001
SGLT2 inhibitor	40,247 (5.05%)	39,933 (5.05%)	280 (6.27%)	34 (2.56%)	<0.001
GLP-1 receptor agonist	45,950 (5.77%)	45,632 (5.77%)	248 (5.55%)	70 (5.27%)	0.60
DPP-4 inhibitor	96,962 (12.18%)	96,377 (12.19%)	426 (9.54%)	159 (11.96%)	<0.001
Thiazolidinedione	38,668 (4.86%)	38,439 (4.86%)	182 (4.08%)	47 (3.54%)	0.004
Other glucose-lowering medication	7,685 (0.96%)	7,629 (0.96%)	41 (0.92%)	15 (1.13%)	0.79

Abbreviations: COPD, chronic obstructive pulmonary disease; DKA, diabetic ketoacidosis; HHS, hyperglycemic hyperosmolar state.

© 2021 McCoy RG et al. *JAMA Network Open*.

eTable 4. Crude and Adjusted Rates of Hyperglycemic Crises Among Patients With Type 1 and Type 2 Diabetes, 2014-2020. Rates adjusted for age (during event), sex, race/ethnicity, U.S. region, and year.

Year	Type 1 Diabetes		Type 2 Diabetes	
	**Crude** N per 1000 PY	**Adjusted** N per 1000 PY	**Crude** N per 1000 PY	**Adjusted** N per 1000 PY
2014	41.75	43.30 (33.37, 53.24)	4.79	4.27 (3.66, 4.88)
2015	47.3	49.07 (40.24, 57.91)	4.00	3.77 (3.40, 4.13)
2016	48.14	49.80 (42.59, 57.01)	3.96	3.81 (3.46, 4.15)
2017	51.26	53.92 (44.62, 63.21)	3.89	3.95 (3.64, 4.25)
2018	53.43	56.77 (48.45, 65.10)	3.81	4.02 (3.76, 4.29)
2019	60.14	61.36 (52.90, 69.82)	3.96	4.29 (4.02, 4.56)
2020	43.52	46.27 (38.59, 53.95)	3.73	4.12 (3.86, 4.38)
Total	50.62	52.69 (48.26, 57.12)	3.70	4.04 (3.88, 4.21)

Abbreviations: DKA, diabetic ketoacidosis; HHS, hyperglycemic hyperosmolar state; PY, person-years

© 2021 McCoy RG et al. *JAMA Network Open*.

eTable 5. Crude and Adjusted Rates of Hyperglycemic Crises Among Patients With Type 1 Diabetes by Prespecified Subgroup, 2014-2020. These data were used to create Figure 1 in the manuscript. Rates adjusted for age (at index date), sex, race/ethnicity, U.S. region.

	**Crude** N per 1000 PY	**Adjusted** N per 1000 PY
**Age group**		
18 - 44 years	66.27	77.57 (68.41, 86.72)
45 - 64 years	39.19	43.54 (36.98, 50.09)
65 - 74 years	36.68	38.82 (28.36, 49.27)
≥75 years	45.60	55.64 (38.13, 73.16)
**Gender**		
Female	62.06	74.91 (66.07, 83.73)
Male	39.57	44.04 (38.59, 49.49)
**Race/ethnicity**		
White	45.69	53.16 (47.69, 58.63)
Black	78.83	96.62 (69.44, 123.81)
Hispanic	56.06	58.93 (45.24, 72.61)
Asian	60.34	66.07 (31.14, 100.99)
**Annual household income**		
<$40,000	84.73	101.52 (82.31, 120.73)
$40,000-$74,999	55.66	65.04 (54.12, 75.96)
$75,000-$124,999	46.06	52.97 (43.94, 62.01)
$125,000-$199,999	34.42	36.22 (28.43, 44.01)
≥$200,000	25.43	26.25 (19.75, 32.75)

Abbreviations: DKA, diabetic ketoacidosis; HHS, hyperglycemic hyperosmolar state; PY, person-years.

© 2021 McCoy RG et al. *JAMA Network Open*.

eTable 6. Crude and Adjusted Rates of Hyperglycemic Crises Among Patients With Type 2 Diabetes by Prespecified Subgroup, 2014-2020. These data were used to create Figure 2 in the manuscript. Rates adjusted for age (at index date), sex, race/ethnicity, U.S. region.

	**Crude** N per 1000 PY	**Adjusted** N per 1000 PY
**Age group**		
18 - 44 years	12.7	13.91 (11.82, 16.00)
45 - 64 years	5.12	5.28 (4.96, 5.60)
65 - 74 years	2.79	2.97 (2.79, 3.14)
≥75 years	2.47	2.70 (2.49, 2.90)
**Gender**		
Female	4.09	4.55 (4.31, 4.79)
Male	3.72	4.04 (3.80, 4.27)
**Race/ethnicity**		
White	3.73	4.15 (3.93, 4.38)
Black	5.80	6.34 (5.87, 6.81)
Hispanic	3.21	3.26 (2.91, 3.60)
Asian	1.44	1.71 (1.31, 2.11)
**Annual household income**		
<$40,000	5.04	5.78 (5.40, 6.17)
$40,000-$74,999	3.65	4.02 (3.75, 4.29)
$75,000-$124,999	3.05	3.11 (2.83, 3.40)
$125,000-$199,999	2.20	2.19 (1.80, 2.58)
≥$200,000	2.36	2.23 (1.70, 2.79)
**Insulin regimen**		
No insulin	1.87	2.00 (1.87, 2.08)
Basal insulin only	7.79	8.35 (7.69, 9.01)
Bolus ± basal insulin	17.3	17.73 (16.53, 18.93)

Abbreviations: DKA, diabetic ketoacidosis; HHS, hyperglycemic hyperosmolar state; PY, person-years.

© 2021 McCoy RG et al. *JAMA Network Open*.

eTable 7. Risk Factors for DKA and HHS (Examined as Independent Outcomes) Among Adults With Type 2 Diabetes, 2014-2020

	DKA		HHS	
	IRR (95% CI)	P-value	IRR (95% CI)	P-value
**Age, years**				
18-44	Ref		Ref	
45-64	0.54 (0.48, 0.60)	<0.001	0.95 (0.73, 1.24)	0.73
65-74	0.39 (0.34, 0.45)	<0.001	0.89 (0.67, 1.17)	0.40
≥75	0.35 (0.30, 0.41)	<0.001	0.92 (0.69, 1.23)	0.57
**Gender**				
Female	Ref		Ref	
Male	0.95 (0.89, 1.03)	0.19	1.17 (1.04, 1.31)	0.008
**Race/Ethnicity**				
White				
Black	1.04 (0.95, 1.15)	0.37	1.59 (1.40, 1.81)	<0.001
Hispanic	0.65 (0.58, 0.73)	<0.001	0.80 (0.67, 0.95)	0.01
Asian	0.55 (0.42, 0.71)	<0.001	0.99 (0.69, 1.42)	0.95
Other/unknown	0.82 (0.67, 1.00)	0.05	1.01 (0.73, 1.41)	0.94
**U.S. census region**				
Midwest	Ref		Ref	
Northeast	0.87 (0.77, 0.99)	0.04	1.07 (0.89, 1.30)	0.46
South	1.04 (0.94, 1.14)	0.44	0.94 (0.82, 1.09)	0.44
West/unknown	1.03 (0.90, 1.19)	0.66	0.88 (0.69, 1.12)	0.29
**Annual household income**				
<$40,000	Ref		Ref	
$40,000-$74,999	0.90 (0.83, 0.99)	0.03	0.70 (0.61, 0.81)	<0.001
$75,000-$124,999	0.80 (0.72, 0.89)	<0.001	0.54 (0.45, 0.64)	<0.001
$125,000-$199,999	0.66 (0.56, 0.78)	<0.001	0.31 (0.23, 0.44)	<0.001
≥$200,000	0.78 (0.62, 0.99)	0.04	0.42 (0.26, 0.65)	<0.001
Unknown	1.06 (0.91, 1.23)	0.48	0.96 (0.76, 1.20)	0.71
**Index year**				
2014	Ref		Ref	
2015	0.90 (0.81, 1.00)	0.05	1.03 (0.88, 1.22)	0.70
2016	0.93 (0.82, 1.05)	0.24	0.92 (0.76, 1.11)	0.38
2017	0.92 (0.82, 1.03)	0.15	1.18 (1.01, 1.40)	0.04
2018	1.00 (0.89, 1.13)	0.95	0.96 (0.80, 1.16)	0.68
2019	1.05 (0.92, 1.20)	0.48	1.02 (0.83, 1.26)	0.86
**Comorbidities**				
Hyperglycemic Crisis	20.32 (17.31, 23.86)	<0.001	7.18 (5.68, 9.06)	<0.001
Severe hypoglycemia	4.49 (3.77, 5.35)	<0.001	3.26 (2.53, 4.20)	<0.001
Retinopathy	1.34 (1.22, 1.47)	<0.001	1.01 (0.97, 1.26)	0.15
Nephropathy	1.29 (1.19, 1.40)	<0.001	1.19 (1.06, 1.34)	0.004
Neuropathy	1.12 (1.03, 1.23)	0.009	1.59 (1.40, 1.80)	<0.0001
Cardiovascular disease	0.92 (0.84, 1.00)	0.05	1.00 (0.88, 1.14)	1.00
Cerebrovascular disease	1.12 (1.00, 1.26)	0.06	1.30 (1.12, 1.50)	<0.001
Peripheral vascular disease	1.06 (0.96, 1.16)	0.28	1.13 (0.99, 1.30)	0.08
Heart failure	1.01 (0.90, 1.14)	0.89	1.41 (1.21, 1.65)	<0.001
Dementia	1.39 (1.14, 1.70)	0.001	1.57 (1.22, 2.01)	<0.001
Hypertension	0.74 (0.67, 0.82)	<0.001	1.60 (1.29, 1.98)	<0.001
Depression	1.48 (1.34, 1.63)	<0.001	1.40 (1.21, 1.62)	<0.001
COPD	1.03 (0.93, 1.15)	0.53	1.35 (1.18, 1.55)	<0.001
Cancer	1.12 (0.98, 1.27)	0.09	1.22 (1.03, 1.46)	0.02
Cirrhosis	1.47 (1.08, 2.01)	0.02	2.71 (1.96, 3.74)	<0.001

© 2021 McCoy RG et al. *JAMA Network Open*.

**Hemoglobin A_{1c}**				
≤5.6%	0.54 (0.39, 0.75)	<0.001	0.59 (0.40, 0.87)	0.008
5.7% – 6.4%	0.63 (0.53, 0.75)	<0.001	0.76 (0.60, 0.96)	0.02
6.5% – 6.9%	Ref		Ref	
7.0% – 7.9%	1.49 (1.29, 1.72)	<0.001	1.47 (1.20, 1.80)	<0.001
8.0% – 8.9%	2.80 (2.41, 3.24)	<0.001	2.06 (1.64, 2.58)	<0.001
9.0% – 9.9%	3.89 (3.31, 4.56)	<0.001	3.12 (2.46, 3.95)	0.008
≥10%	7.22 (6.27, 8.31)	<0.001	6.74 (5.49, 8.27)	<0.001
**Glucose-lowering therapy**				
**No fills**	1.16 (1.02, 1.32)	0.02	1.00 (0.82, 1.23)	0.99
**Insulin use**				
Bolus ± basal	Ref		Ref	
Basal only	0.64 (0.57, 0.71)	<0.001	0.92 (0.79, 1.08)	0.32
Not treated with insulin	0.31 (0.28, 0.35)	<0.001	0.58 (0.49, 0.69)	<0.001
**Non-insulin medications**				
Metformin	0.72 (0.66, 0.78)	<0.001	0.79 (0.69, 0.90)	<0.001
Sulfonylurea	0.88 (0.81, 0.96)	0.006	0.97 (0.84, 1.11)	0.66
SGLT2 inhibitor	1.47 (1.27, 1.70)	<0.001	0.65 (0.46, 0.93)	0.02
GLP-1 receptor agonist	0.74 (0.64, 0.85)	<0.001	0.94 (0.73, 1.20)	0.59
DPP-4 inhibitor	0.84 (0.75, 0.95)	0.004	0.95 (0.80, 1.14)	0.59
Thiazolidinedione	1.18 (0.99, 1.40)	0.07	0.96 (0.72, 1.28)	0.78
Other	0.85 (0.60, 1.20)	0.35	0.83 (0.49, 1.39)	0.47

Abbreviations: COPD, chronic obstructive pulmonary disease; DKA, diabetic ketoacidosis; HHS, hyperglycemic hyperosmolar state.

© 2021 McCoy RG et al. *JAMA Network Open*.
